# Mutations in PINK1 and Parkin Impair Ubiquitination of Mitofusins in Human Fibroblasts

**DOI:** 10.1371/journal.pone.0016746

**Published:** 2011-03-08

**Authors:** Aleksandar Rakovic, Anne Grünewald, Jan Kottwitz, Norbert Brüggemann, Peter P. Pramstaller, Katja Lohmann, Christine Klein

**Affiliations:** 1 Section of Clinical and Molecular Neurogenetics, Department of Neurology, University of Lübeck, Lübeck, Germany; 2 Institute of Genetic Medicine, European Academy, Bolzano, Italy; National Institutes of Health, United States of America

## Abstract

*PINK1* and *Parkin* mutations cause recessive Parkinson's disease (PD). In *Drosophila* and SH-SY5Y cells, Parkin is recruited by PINK1 to damaged mitochondria, where it ubiquitinates Mitofusins and consequently promotes mitochondrial fission and mitophagy.

Here, we investigated the impact of mutations in endogenous PINK1 and Parkin on the ubiquitination of mitochondrial fusion and fission factors and the mitochondrial network structure. Treating control fibroblasts with mitochondrial membrane potential (Δψ) inhibitors or H_2_O_2_ resulted in ubiquitination of Mfn1/2 but not of OPA1 or Fis1. Ubiquitination of Mitofusins through the PINK1/Parkin pathway was observed within 1 h of treatment. Upon combined inhibition of Δψ and the ubiquitin proteasome system (UPS), no ubiquitination of Mitofusins was detected. Regarding morphological changes, we observed a trend towards increased mitochondrial branching in PD patient cells upon mitochondrial stress.

For the first time in PD patient-derived cells, we demonstrate that mutations in PINK1 and Parkin impair ubiquitination of Mitofusins. In the presence of UPS inhibitors, ubiquitinated Mitofusin is deubiquitinated by the UPS but not degraded, suggesting that the UPS is involved in Mitofusin degradation.

## Introduction

Parkinson's disease (PD) is a progressive neurodegenerative disorder, clinically characterized by bradykinesia, tremor, and rigidity, with a monogenic cause in about 2–3% of the cases [1]. Studying the consequences of mutations in recessively inherited PD-associated genes, such as *PTEN-induced putative kinase 1* (*PINK1*) or the E3 ubiquitin ligase *Parkin*, may help to understand the mechanisms underlying the disease also in sporadic, idiopathic PD patients.

Although the exact cause of sporadic PD is still elusive, mitochondrial dysfunction has long been connected with the disease. Impaired respiratory chain function has been found in sporadic PD patients and different *PINK1* or *Parkin* knockdown models [2], [3], [4], [5]. Furthermore, *Drosophila pink1* and *parkin* loss-of-function mutants showed defects in mitochondrial morphology [6], [7], [8], [9], [10]. Transgenic expression of *parkin* markedly ameliorated all *pink1* loss-of-function phenotypes, but not vice versa, suggesting that parkin functions downstream of pink1 [6], [7], [8].

A series of experiments in *Drosophila*, SH-SY5Y cells, and primary mouse neurons provided evidence that the PINK1/Parkin pathway promotes mitochondrial fission and that loss of activity of either protein results in decreased fission and impaired tissue integrity [11], [12]. Inactivation of the dynamin-related protein 1 (drp1), a key factor of mitochondrial fission, enhances the *pink1* and *parkin*-mutant phenotypes in *Drosophila* [11], [12], [13]. By contrast, increased *drp1* gene dosage or inactivation of the mitochondrial fusion-promoting components *optic atrophy 1* (*opa1*) and *mitofusin* (*mfn*) suppress the mitochondrial phenotype in *Drosophila pink1* and *parkin* mutants [11], [12], [13]. Recently, these observations have been linked to mitophagy. Under stress conditions, PINK1 recruits Parkin to dysfunctional mitochondria [14], [15], [16], [17]. The subsequent ubiquitination of Mitofusins by Parkin inhibits mitochondrial fusion and thus promotes mitochondrial fragmentation as an initial step of mitophagy [18], [19], [20].

In PD patient fibroblasts, only the morphological effects of mutations in Parkin have been studied so far revealing that the degree of mitochondrial branching was higher than in controls [21].

In our present work, we used fibroblast cultures from PD patients carrying two mutated *Parkin* or *PINK1* alleles to investigate the consequences of mutations in endogenous PINK1 and Parkin on the ubiquitination of mitochondrial fusion and fission factors. Furthermore, we evaluated the influence of these mutations on the structure of the mitochondrial network in human cells.

## Results

Two fibroblast cultures with homozygous PINK1 mutations, p.Q456X or p.V170G, two cultures with homozygous Parkin mutations, p.V324fsX434 or p.R245fsX253, and fibroblasts from two age-matched mutation-negative healthy controls were included in the study. The effects of these mutations on *PINK1* and *Parkin* mRNA levels are described elsewhere [16], [22]. Clinical features of the mutation carriers were compatible with idiopathic PD, with the exception of an earlier age of onset of 42.3+/−13.5 years [23], [24], [25]. All experiments were performed at least in triplicate and representative blots are shown.

### Decreased Mfn2 levels after valinomycin or CCCP treatment in control fibroblasts

**Figure 1:**
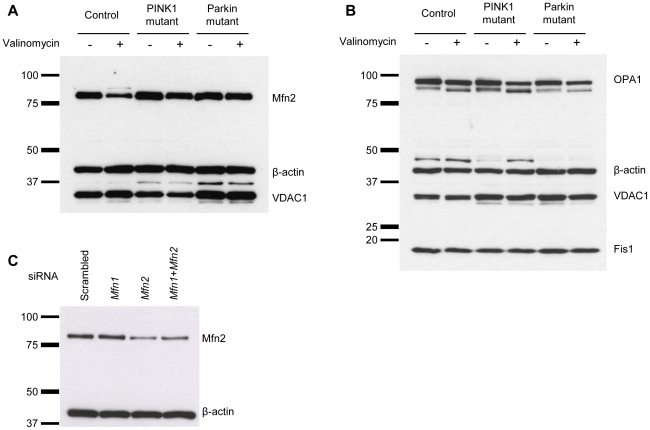
Expression of mitochondrial fusion and fission proteins after valinomycin treatment. Fibroblasts from a healthy control, a homozygous *PINK1* mutant and a homozygous *Parkin* mutant were cultured under basal conditions or treated with 1 µM valinomycin for 12 h. The protein levels of (A) Mfn2, (B) OPA1 and Fis1 were investigated by means of Western blotting. Valinomycin exposure caused a decrease in Mfn2 levels in controls, but not in *PINK1*- or *Parkin*-mutant cells. The protein levels of OPA1 and Fis1 were not influenced by this treatment in any of the cell cultures. Under basal and stress conditions, expression of the mitochondrial marker VDAC1 was comparable in mutants and controls. (C) Mutant cells were incubated with scrambled siRNA, *Mfn1* siRNA, *Mfn2* siRNA or a combination of *Mfn1* and *Mfn2* siRNA for 40 h. Western blot analysis was performed with an antibody against Mfn2. The Mfn2 level decreased only when *Mfn2* siRNA was employed, confirming the specificity of the anti-Mfn1 antibody used in our study. β-actin served as a loading control. Fis1 – fission 1; Mfn1 – mitofusin 1; Mfn2 – mitofusin 2; OPA1 – optic atrophy 1; VDAC1 – voltage-dependent anion channel 1.

First, we determined the endogenous levels of Mfn2 in the *PINK1* and *Parkin* mutants and controls under basal conditions and after exposure to 1 µM valinomycin for 12 h. This treatment caused a drop in the protein levels of Mfn2 in controls but not in either of the mutant cells (Figure 1A). Furthermore in controls, Mfn2 had an additional band on the Western blot, which was about 8 kDa larger in size than the non-modified form, consistent with monoubiquitination of the protein. By contrast, protein levels of OPA1 and Fis1 were unchanged in all cell cultures when incubated with valinomycin (Figure 1B) and modified forms of these proteins were not detectable. Protein levels of the mitochondrial marker voltage-dependent anion channel 1 (VDAC1) were comparable in all samples under basal and stress conditions (Figure 1A, B).

To test whether the used Mfn2 antibody specifically binds Mfn2, a knock-down experiment with siRNA against *Mfn1* or *Mfn2* was performed (Figure 1C). This experiment showed a drop in Mfn2 level only when siRNA against *Mfn2* was employed, confirming the specificity of the antibody.

In this experiment also the Mfn2 homolog mitofusin 1 (Mfn1) [26] was investigated and showed comparable effects (Figure S1). Since the available Mfn1 antibody was less sensitive than the Mfn2 antibody, out of the series of experiments for Mfn2 described in this article, only selected ones were repeated for Mfn1 (see supplementary material).

Reduced Mfn2 levels in controls but not in *PINK1*- and *Parkin*-mutant fibroblasts were also detected after incubation with the protonophore cyanide *m*-chlorophenylhydrazone (CCCP; 10 µM for 12 h) (Figure S2). Since valinomycin and CCCP had identical effects on Mfn2, only results from the experiments using valinomycin are shown.

### In control fibroblasts, Mfn2 is ubiquitinated after valinomycin exposure and is detected in the mitochondrial fraction

**Figure 2:**
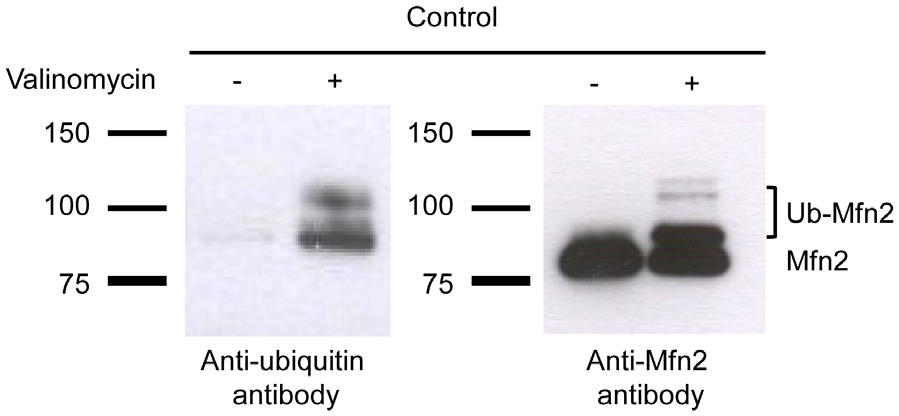
Ubiquitination of Mfn2 upon valinomycin treatment. Fibroblasts from a healthy control were treated with 1 µM valinomycin for 6 h. Whole cell lysates from non-treated and valinomycin treated controls were immunoprecipitated using an antibody against Mfn2. Immunoprecipitates were analyzed by Western blotting using an antibody against ubiquitin (left panel). Subsequently, the membrane was washed and reprobed with an antibody against Mfn2 (right panel). Ubiquitinated forms of Mfn2 (mono- and polyubiquitinated) are present only in valinomycin treated controls. Mfn2 – mitofusin 2; Ub-Mfn2 – ubiquitinated mitofusin 2.

Next, we intended to verify whether the additional band on the Mfn2 blot, which is present only in controls after valinomycin- or CCCP-induced stress, is indeed explained by ubiquitination of the protein. For this, we performed immunoprecipitation using an antibody against Mfn2. Whole cell lysates from non-treated and valinomycin-treated (1 µM, for 6 h) controls were employed. The resulting immunoprecipitates were analyzed by Western blotting with an antibody against ubiquitin (Figure 2, left panel) or with an antibody against Mfn2 (Figure 2, right panel). On both blots, bands of the size of mono- and polyubiquitinated or multiple monoubiquitinated Mfn2 were only detected in valinomycin-treated but not in non-treated controls. Immunoprecipitation was also performed with cell lysates from valinomycin-treated *PINK1*- and *Parkin*-mutant fibroblasts. Western blot analysis with an Mfn2 antibody showed only the non-modified form of the protein (Figure S3). Taken together, these findings supported our previous results and underline that Mfn2 is ubiquitinated via the PINK1/Parkin pathway.

**Figure 3:**
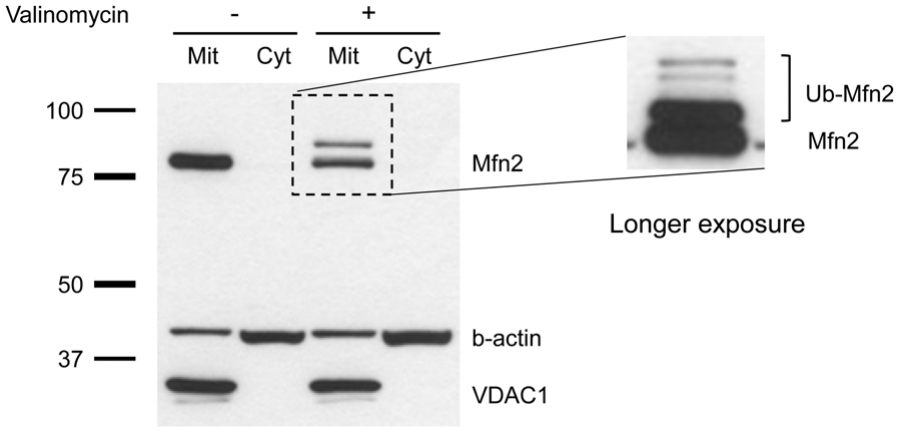
Mitochondrial localization of ubiquitinated Mfn2 after valinomycin treatment. Control fibroblasts were cultured under basal conditions or treated with 1 µM valinomycin for 6 h. Cells were harvested and mitochondrial and cytosolic fractions were analyzed by Western blotting. The subcellular localization of Mfn2 was determined. Quality of cellular fractionation was confirmed using antibodies against VDAC1 and β-actin. The ubiquitinated forms of Mfn2, which were observed only after valinomycin stress, are exclusively found in the mitochondrial fraction. A longer exposure of the blot showed several Mfn2 bands with higher molecular weight, indicative of Mfn2 polyubiquitination (enlarged cutout). Cyt – cytosolic fraction; Mit – mitochondrial fraction; Mfn2 – mitofusin 2; VDAC1 – voltage-dependent anion channel 1.

To determine the subcellular localization of ubiquitinated Mfn1 and 2 in control fibroblasts, cells were incubated with 1 µM valinomycin for 6 h and mitochondrial and cytosolic protein fractions separated. Western blot analysis revealed that Mfn1/2 and their ubiquitinated forms are exclusively localized in the mitochondrial fraction (Figure 3 and Figure S4). The same findings for Mfn2 were obtained when the fractionation experiment was repeated in SH-SY5Y cells (Figure S5).

### Rescue of Mfn2 ubiquitination in mutant fibroblasts

**Figure 4:**
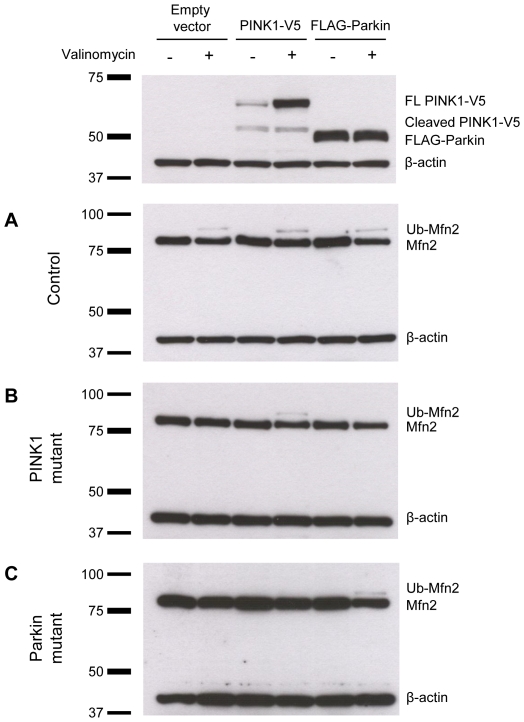
Rescue of Mfn2 ubiquitination. Fibroblasts of a control, a *PINK1* and a *Parkin* mutant were transfected with an empty vector, a vector containing *PINK1-V5* or a vector containing *FLAG-Parkin*. Twenty hours after transfection, cells were cultured under basal conditions or treated with 1 µM valinomycin for an additional 6 h. Western blot analysis using antibodies against V5 and FLAG showed bands of the size of full-length/cleaved PINK1 and Parkin, respectively, in treated and non-treated control cells after transfection, confirming the success of the experiment (upper panel). (A) In controls, ubiquitinated Mfn2 was detected in all three experiments after valinomycin treatment. (B) In *PINK1*-mutant cells, ubiquitination of Mfn2 under stress was rescued through overexpression of *PINK1-V5* but not through overexpression of *FLAG-Parkin*. (C) In *Parkin-*mutant fibroblasts ubiquitinated Mfn2 was only detected after transfection with *FLAG-Parkin*. FL – full-length; Mfn2 – mitofusin 2; Ub-Mfn2 – ubiquitinated mitofusin 2.

To test whether lack of ubiquitination of Mfn2 in the mutants can be rescued, we transfected control, *PINK1*- and *Parkin*-mutant fibroblasts with an empty vector, a vector containing *PINK1-V5* or a vector containing *FLAG-Parkin*. Twenty-four hours after transfection, these cells were cultured under basal conditions or treated with 1 µM valinomycin for an additional 12 h. Whole cell lysates were analyzed by Western blotting. Using antibodies against V5 and FLAG, bands of the size of tagged full-length and cleaved PINK1 or Parkin were detected, confirming successful transfection (Figure 4, upper panel). Furthermore, using an antibody against Mfn2, ubiquitinated Mfn2 was detected in control cells under stress conditions (Figure 4A). In *PINK1*-mutant cells, ubiquitination of Mfn2 under stress was rescued through expression of *PINK1-V5* but not through expression of *FLAG-Parkin* (Figure 4B). Similarly, in *Parkin*-mutant fibroblasts, ubiquitinated Mfn2 was only detected after transfection with *FLAG-Parkin* (Figure 4C).

### Ubiquitination of Mfn2 occurs within 1 h of valinomycin treatment and is prevented in the presence of epoxomicin

**Figure 5:**
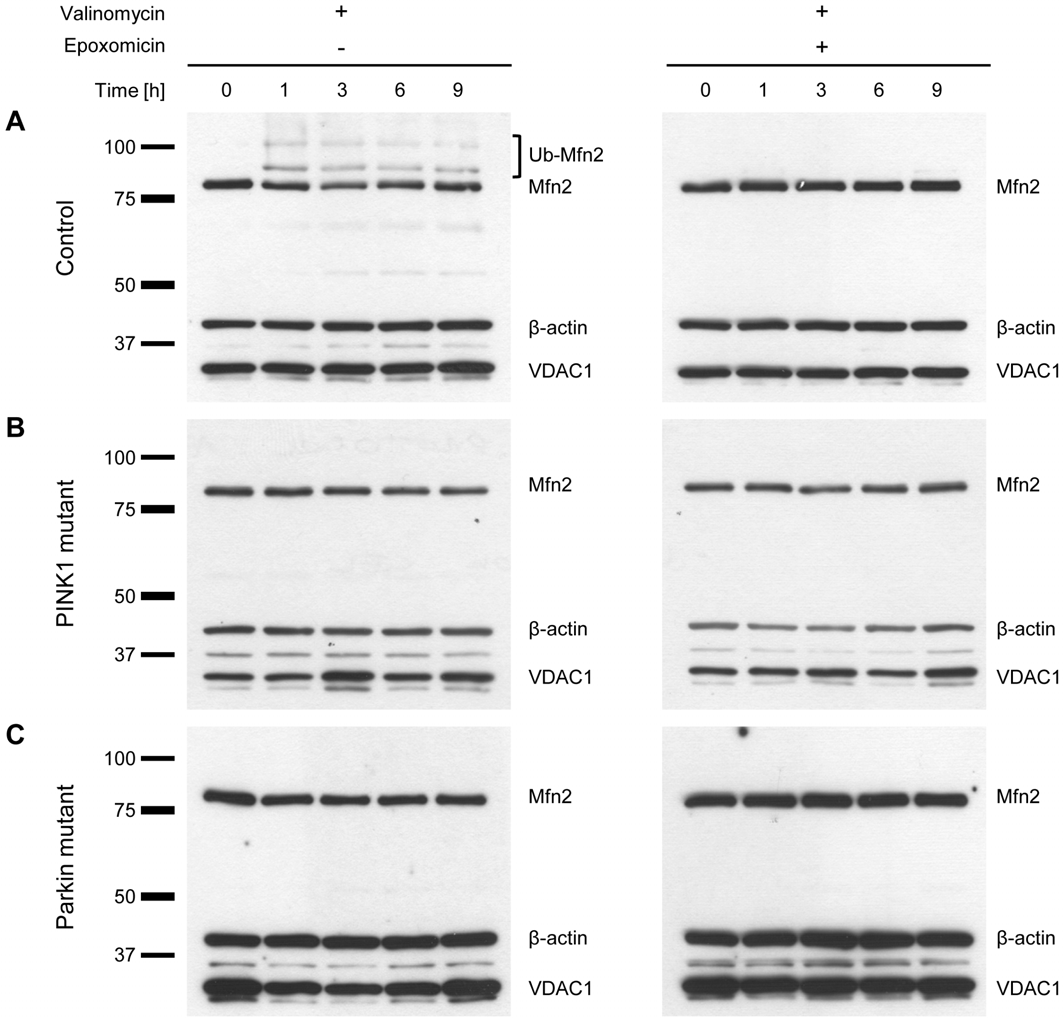
Ubiquitination of Mfn2 is prevented by exposure to epoxomicin. Fibroblasts from (A) a healthy control, (B) a homozygous *PINK1* mutant and (C) a homozygous *Parkin* mutant were treated with 1 µM valinomycin alone (left panel) or with 1 µM valinomycin plus 10 µM epoxomicin (right panel). Proteins were extracted at different time points and analyzed by Western blotting. In control cells, valinomycin treatment initiated the ubiquitination of Mfn2 after 1 h of incubation. This effect was prevented by simultaneous exposure to epoxomicin. The mitochondrial marker VDAC1 and the cytosolic marker β-actin served as loading controls. Mfn2 – mitofusin 2; Ub-Mfn2 – ubiquitinated mitofusin 2; VDAC1 – voltage-dependent anion channel 1.

**Figure 6:**
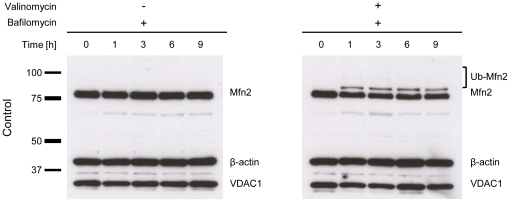
Exposure to bafilomycin has no impact on Mfn2 ubiquitination. Control fibroblasts were treated with 10 nM bafilomycin alone (left panel) or with 10 nM bafilomycin plus 1 µM valinomycin (right panel). Proteins were extracted at different time points and analyzed by Western blotting. Bafilomycin had no effect on the Mfn2 levels and no ubiquitination was detected. When bafilomycin and valinomycin were combined, Mfn2 ubiquitination was initiated by valinomycin and not influenced by bafilomycin. In both experiments, the mitochondrial marker VDAC1 was unchanged. β-actin served as a loading control. Mfn2 – mitofusin 2; Ub-Mfn2 – ubiquitinated mitofusin 2; VDAC1 – voltage-dependent anion channel 1.

To explore whether the ubiquitinated forms of Mfn2 are degraded by the UPS, we treated control (Figure 5A), *PINK1*- (Figure 5B) and *Parkin*-mutant (Figure 5C) fibroblasts with 1 µM valinomycin alone (Figure 5, left panel) or in combination with 10 µM epoxomicin (Figure 5, right panel) and extracted proteins at different time points for Western blot analysis. In control cells, valinomycin treatment initiated the ubiquitination of Mfn2 within 1 h of incubation (Figure 5A, left panel). Mfn2 ubiquitination was prevented by simultaneous exposure to epoxomicin (Figure 5A, right panel). The same effect was observed with MG132 (Figure S6). By contrast, simultaneous treatment with valinomycin and the lysosomal inhibitor bafilomycin did not prevent Mfn2 ubiquitination in control cells (Figure 6). To exclude that proteasomal inhibition influences the effect of the potassium ionophore valinomycin, the mitochondrial membrane potential in control fibroblasts was monitored during 9 h of treatment with valinomycin alone or in combination with epoxomicin. Both culturing conditions caused a similar drop in membrane potential (Figure S7). The protein levels of non-modified Mfn2 remained unchanged in all samples over time when treated with valinomycin plus epoxomicin (Figure 5, right panel). To further test whether epoxomicin alone has an impact on the protein levels of Mfn2, we treated control cells with 10 µM epoxomicin but observed no change in Mfn2 levels during 9 h of incubation (Figure S8).

**Figure 7:**
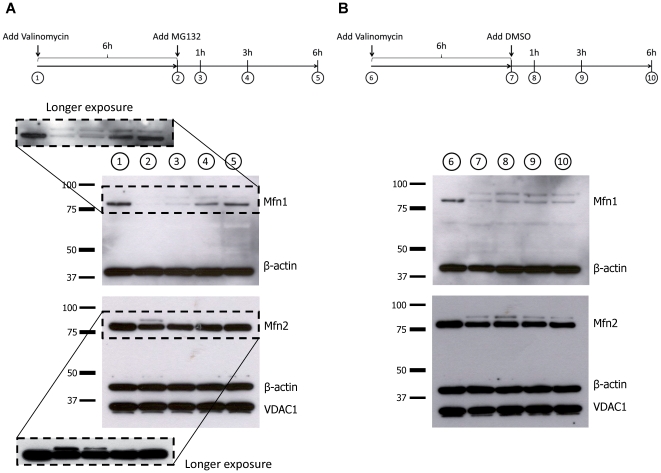
MG132 promotes deubiquitination of Mfn1 and Mfn2. Control fibroblasts were treated with 1 µM valinomycin. After 6 h, MG132 (final concentration 10 µM) was added to the cells. Proteins were extracted at different time points and analyzed by Western blotting. Exposure to (A) an inhibitor of the UPS, i.e.MG132 but not to (B) DMSO alone, induced deubiquitination of both Mfn1 and Mfn2. Mfn1 – mitofusin 1; Mfn2 – mitofusin 2; VDAC1 – voltage-dependent anion channel 1.

Next, we wanted to show that inhibition of the UPS is not only preventing ubiquitination of Mfn1 and Mfn2 (as shown above) but is actually causing deubiquitination of already ubiquitinated Mitofusins. For that we treated control fibroblasts with valinomycin to induce ubiquitination. After 6 h we added MG132 or DMSO (dissolvent for MG132) and harvested cells at different time points. Western blot analysis revealed that upon 6 h of UPS inhibition, levels of non-modified Mitofusins were almost at the same level as before treatment (Figure 7). This additionally confirms that the UPS is involved in the processing of ubiquitinated Mfn1 and Mfn2.

### Exposure to H_2_O_2_ causes ubiquitination of Mfn2

**Figure 8:**
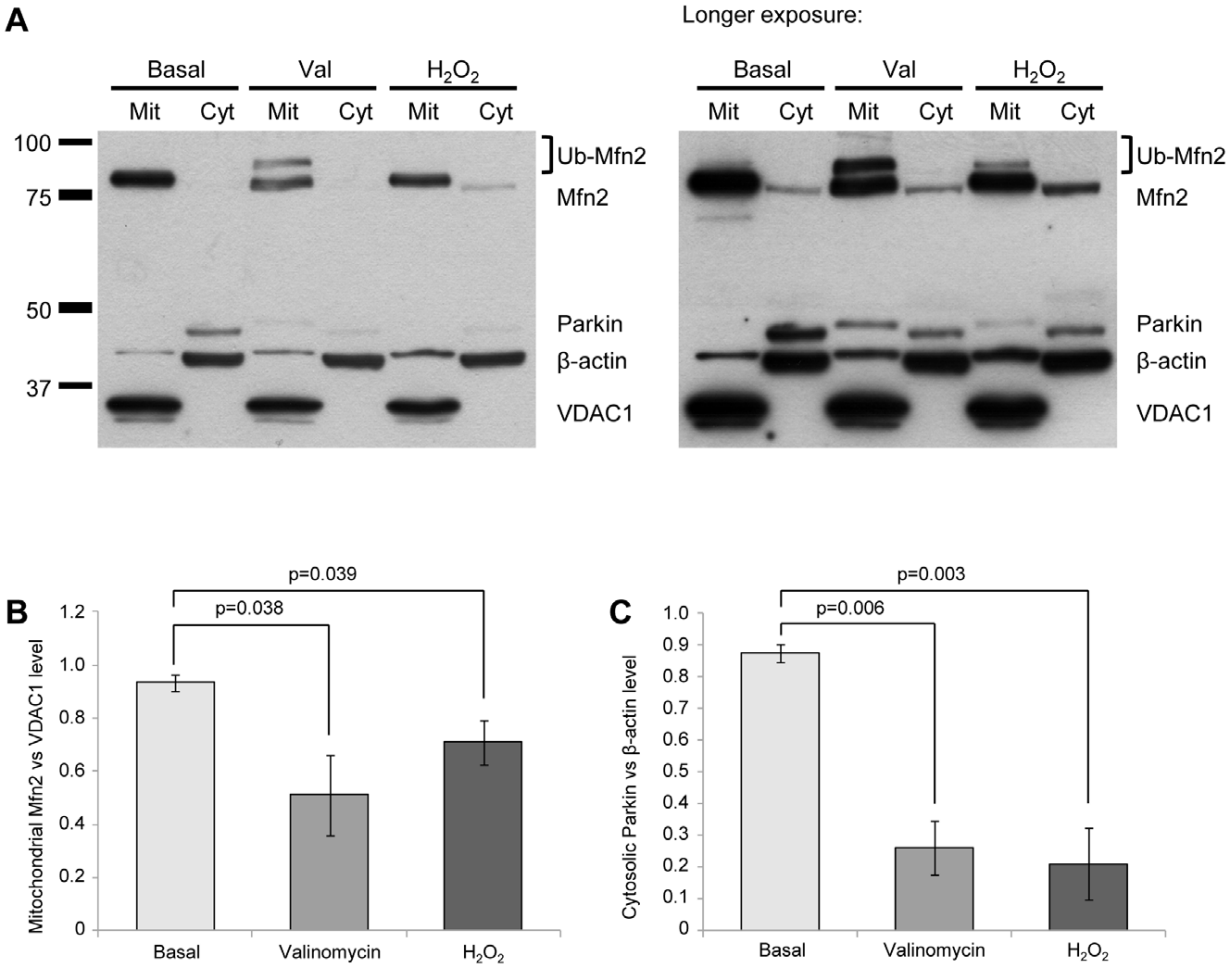
Exposure to H_2_O_2_ causes ubiquitination of Mfn2. Control fibroblasts were cultured under basal conditions, treated with 1 µM valinomycin for 6 h or with 100 µM H_2_O_2_ for 12 h. Cells were harvested and mitochondrial and cytosolic fractions were analyzed by Western blotting. (A) The subcellular localization of Mfn2 and Parkin was determined. Quality of cellular fractionation was confirmed using antibodies against VDAC1 and β-actin. (B) Densitometric analysis of the Western blot results revealed a significant drop in protein levels of non-modified Mfn2 (normalized against VDAC1 expression level) in the mitochondrial fraction after valinomycin or H_2_O_2_ treatment. (C) Furthermore, both treatments caused a significant drop in protein levels of Parkin (normalized against β-actin expression) in the cytosolic fraction. (A, right panel) A longer exposure of the Western blot shows mitochondrial translocation of Parkin after both treatments. For quantification of protein levels, blots of three independent experiments were evaluated. In the graphs mean intensities +/− standard deviation are given. Cyt – cytosolic fraction; H_2_O_2_ – hydrogen peroxide; Mit – mitochondrial fraction; Mfn2 – mitofusin 2; Ub-Mfn2 – ubiquitinated mitofusin 2; Val – Valinomycin; VDAC1 – voltage-dependent anion channel 1.

Next, we investigated whether exposure of control fibroblasts to the superoxide generator H_2_O_2_ also results in ubiquitination of Mfn2. Therefore, cells were incubated with 100 µM H_2_O_2_ for 12 h and compared to cells stressed with 1 µM valinomycin for 6 h and non-treated cells. Mitochondrial and cytosolic fractions of these cells were analyzed by Western blotting using antibodies against Mfn2, Parkin, VDAC1 and β-actin. The predominant presence of VDAC1 in the mitochondrial and of β-actin in the cytosolic fraction indicated good quality of the fractionation (Figure 8A). Densitometric analysis revealed a significant drop in protein levels of non-modified Mfn2 in the mitochondrial fraction after valinomycin but also after H_2_O_2_ treatment (Figure 8A and B). Under both stress conditions, high-molecular-weight bands of Mfn2 were detected, indicative of Mfn2 ubiquitination. As already demonstrated in our recently published study on *PINK1*- and *Parkin*-mutant fibroblasts [16], both treatments caused a significant drop in protein levels of Parkin in the cytosolic fraction (Figure 8A and C). Longer exposure of the Western blots revealed mitochondrial translocation of endogenous Parkin after both treatments (Figure 8A, right panel).

### Branching of the mitochondrial network

**Figure 9:**
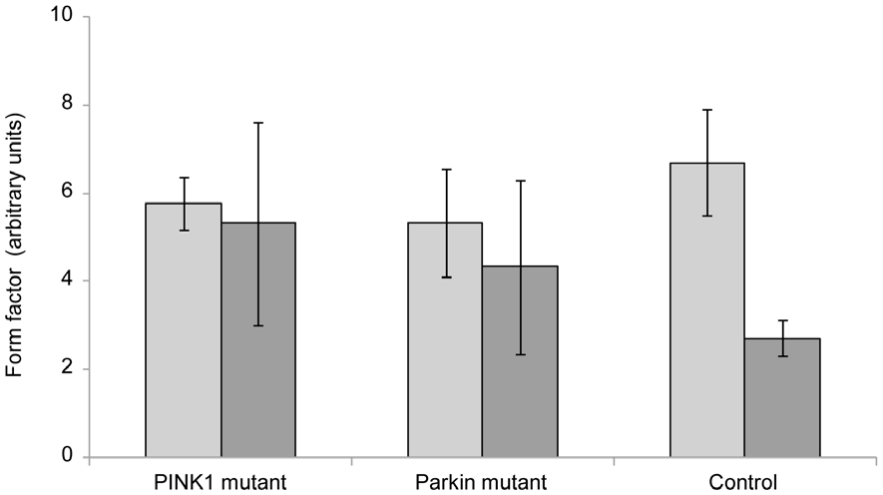
Branching of mitochondrial network. The mitochondrial form factor was determined in a control, a homozygous *PINK1* and a homozygous *Parkin* mutant. The quantification revealed no differences in mitochondrial branching between mutant and control cells under basal conditions (light gray bars). After treatment with 1 µM valinomycin for 12 h, a decrease in branching occurred in all individuals, with the control being most severely affected (dark gray bars). In the graph, mean form factors +/− standard deviation are given.

Finally, we determined the impact of the absence of Mfn2 ubiquitination in the mutants under stress conditions on the mitochondrial network. To evaluate the degree of mitochondrial branching, we measured the form factor [21] in cells from a *PINK1* mutant, a *Parkin* mutant and a control. This showed a comparable degree of mitochondrial network branching in all investigated individuals under basal conditions. When we stressed the cells with 1 µM valinomycin for 12 h to initiate Mfn2 ubiquitination, the form factor decreased in all samples. Although there was a trend towards more fragmented mitochondria in control than in mutant cells, this difference did not reach significance (Figure 9).

## Discussion

Mitochondrial dysfunction and changes in mitochondrial morphology have long been linked to the disease mechanisms underlying PD [2], [4], [5], [6], [7], [27]. However, only recently, several studies demonstrated that the various observed mitochondrial phenotypes can be ascribed to one common molecular cause: Apparently, a deficit in mitophagy leads to accumulation of dysfunctional mitochondria in the cell [14], [15], [16], [17]. The PD-associated proteins PINK1 and Parkin seem to play a central role in the initiation of mitophagy [18], [19], [20], [28]. In a recent study, we have established human fibroblasts with homozygous *PINK1* and *Parkin* mutations as a suitable model system to investigate the PINK1/Parkin pathway [16]. Here, we expand our previous results using these PD patient cells to characterize effects of the PINK1/Parkin pathway on mitochondrial fusion and fission proteins on the endogenous level.

Several studies reported that mitochondrial stress, such as exposure to membrane potential inhibitors, initiates the PINK1/Parkin mitophagy pathway [14], [15], [16], [17], [20], [28]. Therefore, we treated our fibroblast cell cultures with the mitochondrial uncoupling agents valinomycin and CCCP or the superoxide generator H_2_O_2_. All treatments resulted in decreased Mfn2 signal in the controls but not in the mutants. However, the effect after H_2_O_2_ incubation was the least pronounced. Moreover, high-molecular-weight Mfn2 bands were detected in the controls, indicative of Mfn2 poly- or multiple monoubiquitination. By contrast, the protein levels of OPA1 and Fis1 were not altered in mutants compared to controls under stress conditions. In *Drosophila*, the mitochondrial phenotype caused by *pink1* and *parkin* loss-of-function mutations could at least partially be suppressed by *opa1* knockdown [8], [11], [12], [13]. Conversely, in SH-SY5Y cells, overexpression of *OPA1* prevented changes in mitochondrial morphology induced by *PINK1* or *Parkin* knockdown. However, similar to our findings, no alterations in OPA1 processing were observed in these cells lacking PINK1 or Parkin [29]. These apparent discrepancies could be explained by differences in OPA1 function in arthropods compared to mammals [30].

By means of immunoprecipitation, the additional anti-Mfn2 reactive bands indeed proved to represent ubiquitinated forms of the protein. These findings are in line with recent publications reporting that in wild-type *Drosophila* and SH-SY5Y cells Mitofusins are ubiquitinated in response to mitochondrial stress. This modification was, however, impaired in treated *Parkin* or *Pink1* knockdown cells [18], [19], [20], [28]. Furthermore, studies comparing wild-type flies with *parkin* or *pink1* null mutants suggested that loss of parkin or pink1 increases the steady-state abundance of mfn [18], [19]. We did not detect any changes in protein levels when monitoring Mfn2 in *PINK1*- or *Parkin*-mutant human fibroblasts under stress conditions over time. Therefore, it is tempting to speculate that the “increased” mfn levels in *pink1* or *parkin* knockdown flies may reflect an increase in mitochondrial biogenesis in these mutants [17].

Of note, all experiments performed for the Mfn2 interaction partner Mfn1 in this study indicate similar behavior of both Mitofusins in the PINK1/Parkin pathway.

Furthermore, we were able to rescue the ubiquitination of Mfn2 when *PINK1* or *Parkin* was re-expressed in *PINK1*- or *Parkin*-mutant cells. In *Drosophila*, transgenic expression of *parkin* in pink1 loss-of-function mutants markedly ameliorated all mitochondrial phenotypes, but not vice versa, leading to the conclusion that parkin functions downstream of pink1 [6], [7], [8]. However, to our surprise, we were not able to detect ubiquitinated Mfn2 when *PINK1* mutant cells were transfected with *FLAG-Parkin*. Given the weak signal of ubiquitinated Mfn2 after *PINK1* transfection in the *Parkin*-mutant cells, a possible explanation for this discrepancy might be that the used antibody is not sensitive enough to detect the likely even lower levels of ubiquitinated Mfn2 in the *PINK1*-mutant fibroblasts.

**Figure 10:**
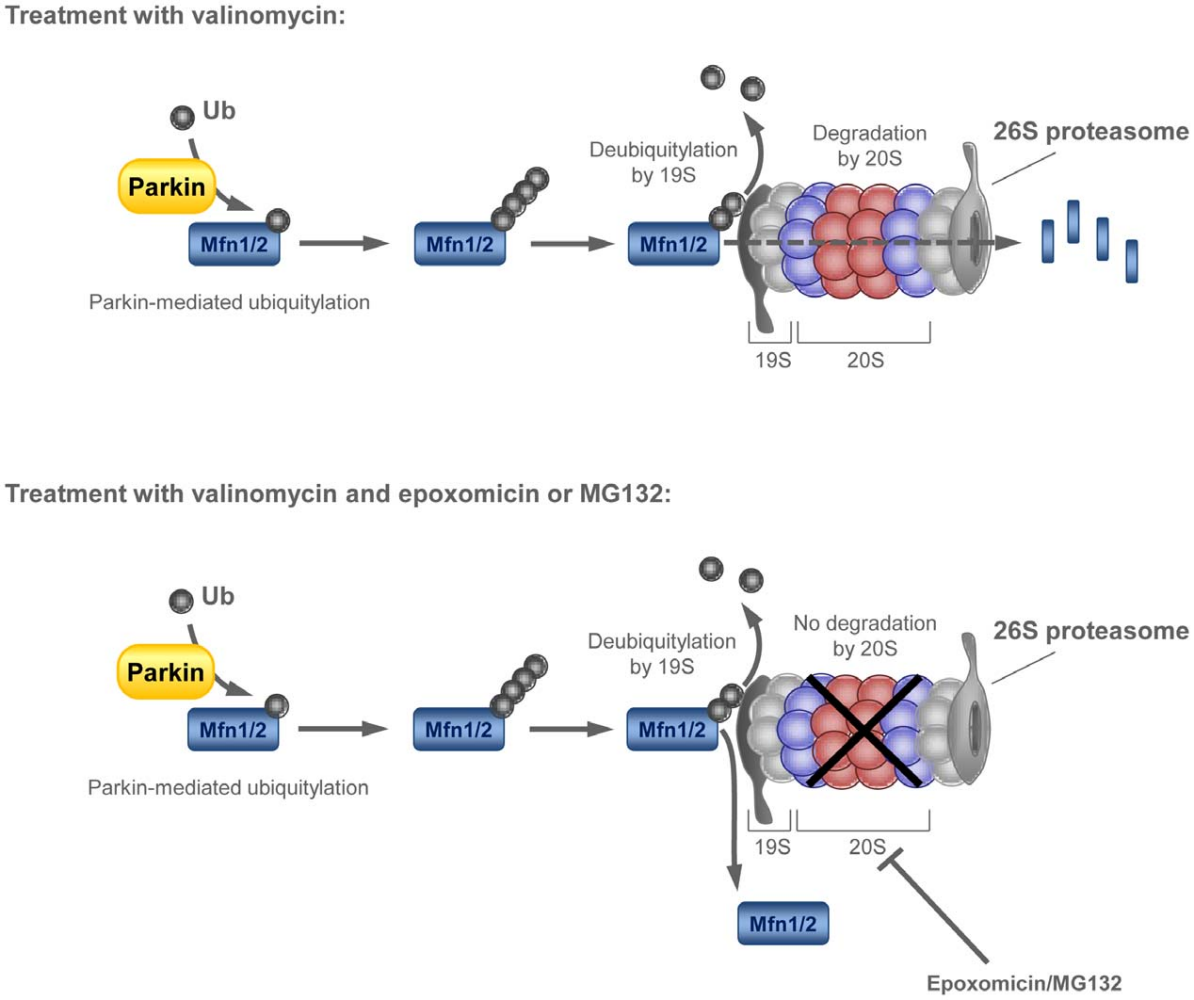
Possible scenario of Mfn1/2 deubiquitination and degradation at the UPS. Under valinomycin stress, Mitofusins are ubiquitinated by Parkin. Poly-ubiquitinated Mitofusins are subsequently recognized by the intrinsic ubiquitin-binding activity of the 19S particle of the 26S proteasome. At the 19S regulatory complex the ubiquitin chain is disassembled, and the substrate is unfolded before it can enter the cavity of the 20S subunit where proteolysis takes place [31]. Simultaneous treatment with epoxomicin (or MG132) inhibits the degradation function of the 20S core particle but does not influence the deubiquitylase activity of the 19S subunit. Consequently, poly-ubiquitinated Mitofusins are deubiquitinated at the proteasome but cannot be degraded. Mfn1/2 – mitofusin 1/2; Ub – ubiquitin; UPS – ubiquitin proteasome system.

When we determined the subcellular localization of non-modified and ubiquitinated Mfn2 in control fibroblasts and SH-SY5Y cells, all Mfn2 forms were exclusively found in the mitochondrial fraction. Ubiquitination occurred already within one hour of treatment with valinomycin. Contrary to our expectations, inhibition of the UPS with epoxomicin or MG132 neither increased nor preserved the levels of ubiquitinated Mfn2 over time. Ubiquitination of Mfn2 in control cells was apparently absent using valinomycin in combination with a proteasomal inhibitor. However, this was not explained by interference of epoxomicin with the effect of the mitochondrial uncoupler valinomycin. According to the literature, proteins targeted for degradation can only enter the UPS after their ubiquitin chain has been removed. This deubiquitination is performed by the 19S regulatory complex of the UPS [31], [32]. Epoxomicin is a potent inhibitor of the 20S proteasome subunit, where protein degradation takes place, but does not influence the deubiquitinase activity of the 19S particle (Figure 10) [33]. Consequently, our data suggest that, in the presence of epoxomicin or MG132, ubiquitinated Mitofusins are solely deubiquitinated but not degraded by the UPS leading to constant levels of non-modified Mfn1/2 in the stressed control cells. Under stress conditions without UPS inhibition, however, the turnover of ubiquitinated Mfn1/2 in the cytosol is probably occurring too rapidly to be detected in a fractionation experiment. In line with our hypothesis, inhibition of lysosomal degradation did not prevent Mfn2 ubiquitination in control fibroblasts. At first sight, there appears to be a discrepancy between our data and a recently published report showing that the ubiquitinated forms of Mitofusins may be preserved upon treatment with the UPS inhibitor MG132 and the uncoupler of the mitochondrial membrane potential CCCP [28]. However, this can be explained by the fact that cells overexpressing *Parkin* were used in that study, whereas our experimental setup was based on endogenous levels of Parkin. It is conceivable that under conditions of overexpressed *Parkin*, the balance between Parkin-mediated ubiquitination and deubiquitination by the UPS is shifted towards ubiquitination, thus leading to accumulation of ubiquitinated Mitofusins in their system. Interestingly, the degradation of the yeast Mfn1/2 homologue Fzo1 is also dependent on the UPS [34].

**Figure 11:**
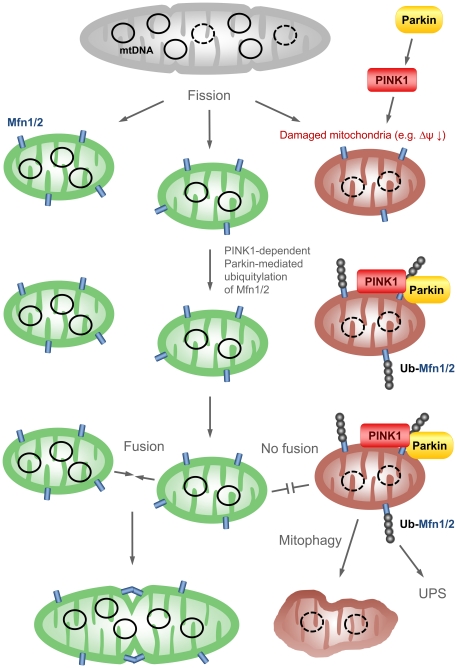
Scheme summarizing PINK1's and Parkin's putative function in mitophagy. By means of fission, mitochondria are randomly divided. Damaged mitochondria can be distinguished from functional mitochondria, for instance, by a difference in membrane potential. Dysfunctional mitochondria with low membrane potential are detected by PINK1, which recruits Parkin. Next, Parkin ubiquitinates Mfn1/2 which localize to the outer mitochondrial membrane. Subsequently, ubiquitinated Mitofusins are degraded by the UPS, preventing fusion of dysfunctional with functional mitochondria. By this, dysfunctional mitochondria are selected out from the general pool of mitochondria and subsequently undergo mitophagy. Δψ – mitochondrial membrane potential; Mfn1/2 – mitofusin 1/2; PINK1 – PTEN-induced putative kinase 1; Ub-Mfn1/2 – ubiquitinated mitofusin 1/2; UPS – ubiquitin proteasome system.

In a recent publication, the mitochondrial membrane potential was identified as an important cellular parameter to differentiate between functional and dysfunctional mitochondria. Following fission, the refusion of daughter mitochondria requires a membrane potential beyond a certain threshold [35]. We hypothesize that Parkin-mediated ubiquitination and subsequent degradation of Mfn1/2 prevents this refusion. Such isolated dysfunctional mitochondria likely undergo mitophagy and require both functional Parkin and PINK1. This notion is supported by colocalization of PINK1 and partially also of Parkin with microtubule-associated protein 1 light chain 3 (LC3), a marker of autophagosomes [36], and by abrogation of Parkin-induced mitophagy upon treatment with bafilomycin, a lysosomal inhibitor [37]. For a schematic representation of the putative Parkin/PINK1 mitophagy pathway, see Figure 11.

According to our current knowledge, mitophagy is the only mechanism by which mitochondria are recycled [38]. Therefore, impairment of mitophagy due to PINK1 or Parkin mutations presumably leads to accumulation of dysfunctional mitochondria in the cell. This scenario may be an explanation for mitochondrial phenotypes, such as respiratory chain dysfunction [3], [4], [21] and elevated mitochondrial DNA mutational load [39], [40], [41] which have been observed in PINK1 or Parkin knockout models as well as PD patients with mutations in either gene (Figure 11). In accordance with our hypothesis, two studies in HeLa cells provided evidence that overexpression of Parkin leads to a significant loss of mitochondria [36], [37]. However, when we compared the expression of various mitochondrial markers in our *PINK1*- and *Parkin*-mutant as well as control fibroblasts, no changes indicative of differences in mitochondrial mass were identified. A possible explanation for this discrepancy is that mitophagy is highly selective in an endogenous model and thus does not result in a readily observable reduction in mitochondrial mass [38], [42].

To obtain insight in the consequences of altered Mfn1/2 ubiquitination on mitochondrial morphology, we determined the degree of branching of the mitochondrial network by measuring the form factor [21] in *PINK1*-, *Parkin*-mutant and control cells. In line with our qualitative observations, this quantitative assessment showed no differences between mutant and control fibroblasts under basal conditions. By contrast, mitochondrial branching was found to be significantly increased in non-treated *Parkin*-mutants in an earlier study on fibroblasts [21]. When we stressed the mutant and control cells with valinomycin, the form factor decreased in all three samples. However, we detected a trend towards more fragmented mitochondria in control cells, supporting our hypothesis. It will be interesting to see whether this trend holds up in a larger sample of control, *PINK1*- and *Parkin*-mutant fibroblast cultures. In the above-mentioned study [21], fibroblasts were exposed to rotenone, an inhibitor of the respiratory chain complex I. This treatment caused mitochondrial fragmentation in *Parkin*-mutant and control cells similar to the effect of valinomycin in our cells. In this published study, however, no differences in branching were detected between the investigated groups after exposure to mitochondrial stress [21]. Since mitochondrial fusion and fission are transient events, dynamic quantification methods would be useful to determine the impact of PINK1 and Parkin mutations on mitochondrial morphology.

Confirming the results from arthropod studies and expanding on our previous findings, we showed that endogenous mutations in PINK1 and Parkin impair ubiquitination of mitofusins in human fibroblasts. In addition, our results imply that the UPS is involved in the degradation of Mitofusins under mitochondrial stress conditions.

## Materials and Methods

### Ethics statement

Written informed consent was obtained from all study participants and the study was approved by the local ethics committee of the University of Lübeck.

### Tissue culture

Human dermal primary fibroblasts used in the present study were described before [16], [22], [43]. Fibroblasts and commercially available SH-SY5Y were cultured in high glucose Dulbecco's Modified Eagle's Medium supplemented with 10% fetal bovine serum and 1% penicillin–streptomycin (all PAA) at 37°C, 5% CO_2_. In all assays, fibroblast passage numbers were matched (<10).

To induce mitochondrial stress, fibroblasts and SH-SY5Y cells were treated with the potassium ionophore valinomycin (1 µM, Sigma), the protonophore CCCP (10 µM, Sigma) or with the superoxide generator H_2_O_2_ (100 µM, Sigma). For inhibition of the proteasome system epoxomicin (10 µM, Sigma) or MG132 (10 µM, Sigma) were used. To inhibit the acidification of lysosomes bafilomycin was employed (10 nM, Sigma).

### Mitochondrial preparation

Mitochondria were isolated from fibroblasts as previously described [43]. In brief, cells were harvested and homogenized in buffer containing 250 mM sucrose, 10 mM Tris and 1 mM EDTA, pH 7.4. After that, nuclei and unbroken cells were removed by centrifugation at 1,500×g for 20 min. The supernatant containing intact mitochondria was transferred into a new tube and centrifuged at 12,000×g for 10 min. Supernatant (“cytosolic fraction”) was transferred into another new tube and the mitochondria-enriched pellet (“mitochondrial fraction”) was dissolved in radioimmunoprecipitation assay (RIPA) buffer containing a cocktail of protease and phosphatase inhibitors (Roche Diagnostics).

Cytoplasmic fractions were concentrated by using centricon YM-10 devices (Millipore) according to the manufacturer's instructions. Proteins of the mitochondrial and cytoplasmic fractions were separated by sodium dodecyl sulfate polyacrylamide gel electrophoresis (SDS PAGE) and detected by Western blot analysis using appropriate antibodies.

### Protein extraction

Proteins were extracted using RIPA buffer containing 0.1% SDS (50 mM Tris-HCl pH 7.6, 150 mM NaCl, 1% DOC, 1% NP-40 and 0.1% SDS). Cells or mitochondria-enriched pellets were dissolved in the appropriate amount of buffer and incubated on ice for 30 min. After that, the lysates were centrifuged at 16,000×g for 20 min at 4°C. The supernatant was transferred into a new tube and used for Western blotting.

### Immunoprecipitation

Fibroblasts plated in 15 cm Petri dishes were treated with 1 µM valinomycin. Next, cells were harvested and resuspended in 1 ml of lysis buffer (150 mM NaCl, 50 mM Tris-HCl pH 7.6, 1% NP-40, 0.1% SDS, protease inhibitor cocktail (Roche Diagnostics)). Lysates were incubated on ice for 30 min and cleared by centrifugation at 13,000×g for 10 min. Samples were equalized for the protein concentration and incubated with 5 µl of anti-Mfn2 antibody overnight on a rotator. Fifty µl of washed protein G agarose beads (Roche Diagnostics) were added to the samples. This was followed by incubation for 2 h on a rotator. Next, the beads were pelleted by centrifugation and the supernatant was discarded. The beads were washed three times with lysis buffer followed by resuspension into 2× loading buffer (Invitrogen) and incubation at 95°C for 5 min. After centrifugation, the supernatant was analyzed by Western blotting.

### Western blot analysis

SDS PAGE was performed using NuPAGE 4–12% Bis-Tris gels (Invitrogen). After electrophoresis, proteins were transferred to the nitrocellulose membrane (Protran) and probed with antibodies raised against Mfn1 (Abcam, # ab60939), Mfn2 (Abcam, # ab56889), β-actin (Sigma, # A 5316), VDAC1 (Abcam, # ab14734), OPA1 (Abcam, # ab42364), Fis1 (Alexis Biochemicals, # ALX-210-907), FLAG M2 (Sigma, # F 1804), V5 (Invitrogen, # R960-25) and ubiquitin (Boston Biochem, # AB-001). All Western blot analyses were performed in triplicates for all available mutants (PINK1: p.Q456X and p.V170G; Parkin: p.V324fsX434 and p.R245fsX253) and two controls, and representative blots are shown in the figures. For densitometric analyses TotalLab software (Nonlinear Dynamics) was used.

### Transient transfection

Fibroblasts were transiently transfected with *pcDNA3.1 V5/His* (Invitrogen) containing full-length wild-type *PINK1* cDNA (FL PINK1). For overexpression of *Parkin*, N-terminally *FLAG*-tagged full-length *Parkin* cDNA was cloned in pcDNA3.1 (Invitrogen's modified vector lacking V5/His tags). For *Mfn1* or *Mfn2* knock-down, Hs_MFN1_5 or Hs_MFN2_5 validated siRNAs (both Qiagen) (final concentration 50 nM) were used and scramble siRNA (Silencer negative control 1 siRNA [Ambion]) (final concentration 50 nM) with no known mammalian homology served as negative control. All transfections of fibroblasts were performed using the Nucleofector Device (Lonza).

### Analysis of the mitochondrial membrane potential

The mitochondrial membrane potential was analyzed using 5,5′,6,6′-tetrachloro-1,1′,3,3′-tetraethylbenzimidazolylcarbocyanine iodide (JC-1; Invitrogen) according to the manufacturer's protocol [44].

### Assessment of mitochondrial branching

The mitochondrial network in fibroblasts was stained with an anti-GRP75 antibody (Abcam, # ab53098) in combination with the zenon immunolabelling kit (Invitrogen) according to the manufacturer's protocol.

The morphology of the mitochondrial network was investigated using a fluorescence microscope equipped with an ApoTome and AxioVision software (all Zeiss). By means of ImageJ 1.42, raw images were binarized, mitochondrion area and outline were measured and the form factor was calculated [21]. Images of at least five randomly selected cells per individual were analyzed under basal conditions and after treatment with valinomycin.

### Statistical analysis

For evaluation of the impact of stress on cells, a paired Student's t-test was used to determine differences before and after treatment. All p-values below 0.05 were considered indicative of a significant difference between measurements and are shown by an asterisk.

## Supporting Information

Figure S1 **Protein levels of Mfn1 after valinomycin treatment.** (A) Fibroblasts from a healthy control, a homozygous *PINK1* mutant and a homozygous *Parkin* mutant were cultured under basal conditions or treated with 1 µM valinomycin for 12 h. The protein levels of Mfn1 were investigated by means of Western blotting. Valinomycin exposure caused a drop in Mfn1 levels in controls, but not in *PINK1*- or *Parkin*-mutant cells. β-actin served as a loading control. (B) Mutant cells were transfected with scrambled siRNA, *Mfn1* siRNA, *Mfn2* siRNA or a combination of *Mfn1* and *Mfn2* siRNA for 40 h. Western blot analysis was performed with an antibody against Mfn1. The Mfn1 levels decreased only when *Mfn1* siRNA was employed, confirming the specificity of the anti-Mfn1 antibody used in our study. Mfn1 – mitofusin 1; Mfn2 – mitofusin 2.
(TIFF)

Figure S2 **Protein levels of Mfn2 after CCCP treatment.** Fibroblasts from two healthy controls, two homozygous *PINK1* mutants and one homozygous *Parkin* mutant were cultured under basal conditions or treated with 10 µM CCCP for 12 h. The protein levels of Mfn2 were investigated by means of Western blotting. CCCP exposure caused a decrease in Mfn2 levels in controls, but not in *PINK1*- or *Parkin*-mutant cells. β-actin served as a loading control. CCCP – cyanide *m*-chlorophenylhydrazone; Mfn2 – mitofusin 2.
(TIFF)

Figure S3 **Immunoprecipitation with antibodies against Mfn2.** Control, *PINK1*- and *Parkin*-mutant cells were treated with 1 µM valinomycin for 12 h. Cells were harvested and whole cell lysates were used for immunoprecipitation with antibodies against GRP75 or Mfn2. The resulting precipitates were analyzed by Western blotting using antibodies against Mfn2 and GRP75. An Mfn2 immunoreactive band of higher molecular weight was detected only in controls but not in either of the mutants. Immunoprecipitation with an antibody against the mitochondrial marker GRP75 served as a negative control. GRP75 – glucose-regulated protein 75; IgG – immunoglobulin G; Mfn2 – mitofusin 2; Ub-Mfn2 – ubiquitylated mitofusin 2.
(TIFF)

Figure S4 **Mitochondrial localization of ubiquitylated Mfn1 and Mfn2 after valinomycin treatment.** Control fibroblasts were cultured under basal conditions or treated with 1 µM valinomycin for 6 h. Cells were harvested, proteins of mitochondrial and cytosolic fractions were loaded on two SDS-PAGE gels and analyzed by Western blotting. The subcellular localizations of Mfn1 and Mfn2 were determined. Quality of cellular fractionation was confirmed using antibodies against VDAC1 and β-actin. The ubiquitylated forms of Mfn1 and Mfn2, which were observed only after valinomycin stress, are exclusively found in the mitochondrial fraction. Cyt – cytosolic fraction; Mit – mitochondrial fraction; Mfn1 – mitofusin 1; Mfn2 – mitofusin 2; VDAC1 – voltage-dependent anion channel 1.
(TIFF)

Figure S5 **Mitochondrial localization of ubiquitylated Mfn2 after valinomycin treatment in SH-SY5Y cells.** SH-SY5Y cells were cultured under basal conditions or treated with valinomycin for 6 h. Cells were harvested and mitochondrial and cytosolic fractions were analyzed by Western blotting. The subcellular localization of Mfn2 was determined. Quality of cellular fractionation was confirmed using antibodies against VDAC and β-actin. The ubiquitylated forms of Mfn2, which were observed only after valinomycin stress, are exclusively found in the mitochondrial fraction. Mfn2 – mitofusin 2; Ub-Mfn2 – ubiquitylated mitofusin 2; VDAC1 – voltage-dependent anion channel 1.
(TIFF)

Figure S6 **Ubiquitylation of Mfn2 occurs within 1 h of valinomycin treatment.** Fibroblasts from a healthy control were treated with 1 µM valinomycin alone (left panel) or with 1 µM valinomycin plus 10 µM MG132 (right panel). Proteins were extracted at different time points and analyzed by Western blotting. Valinomycin treatment initiated the ubiquitylation of Mfn2 after 1 h of incubation. This effect was prevented by simultaneous exposure to MG132. β-actin served as a loading control. An unspecific band is marked by an asterisk. Mfn2 – mitofusin 2; Ub-Mfn2 – ubiquitylated mitofusin 2.
(TIFF)

Figure S7 **Treatment with valinomycin alone or in combination with epoxomicin causes a drop in mitochondrial membrane potential.** Control fibroblasts were incubated with either 1 µM valinomycin alone or with 1 µM valinomycin plus 10 µM epoxomicin. The membrane potential was measured at different time points and corrected for protein concentration. Exposure to the proteasome inhibitor epoxomicin did not influence the membrane potential over time. In the graph mean values +/− standard deviation of three independent experiments are given. Δψ – mitochondrial membrane potential.
(TIFF)

Figure S8 **No accumulation of Mfn2 after exposure to epoxomicin.** Control fibroblasts were treated with 10 µM epoxomicin. Proteins were extracted at different time points and analyzed by Western blotting. Exposure to the proteasome inhibitor epoxomicin did not affect the expression of Mfn2 over time. The mitochondrial marker VDAC1 and the cytosolic marker β-actin served as loading controls. Mfn2 – mitofusin 2; VDAC1 – voltage-dependent anion channel 1.
(TIFF)
