# Supplementary figures and images for: Mutations in PINK1 and Parkin Impair Ubiquitination of Mitofusins in Human Fibroblasts
Source: PLoS One. 2011 Mar 8;6(3):e16746. doi: 10.1371/journal.pone.0016746 (PMC3050809; doi:10.1371/journal.pone.0016746)

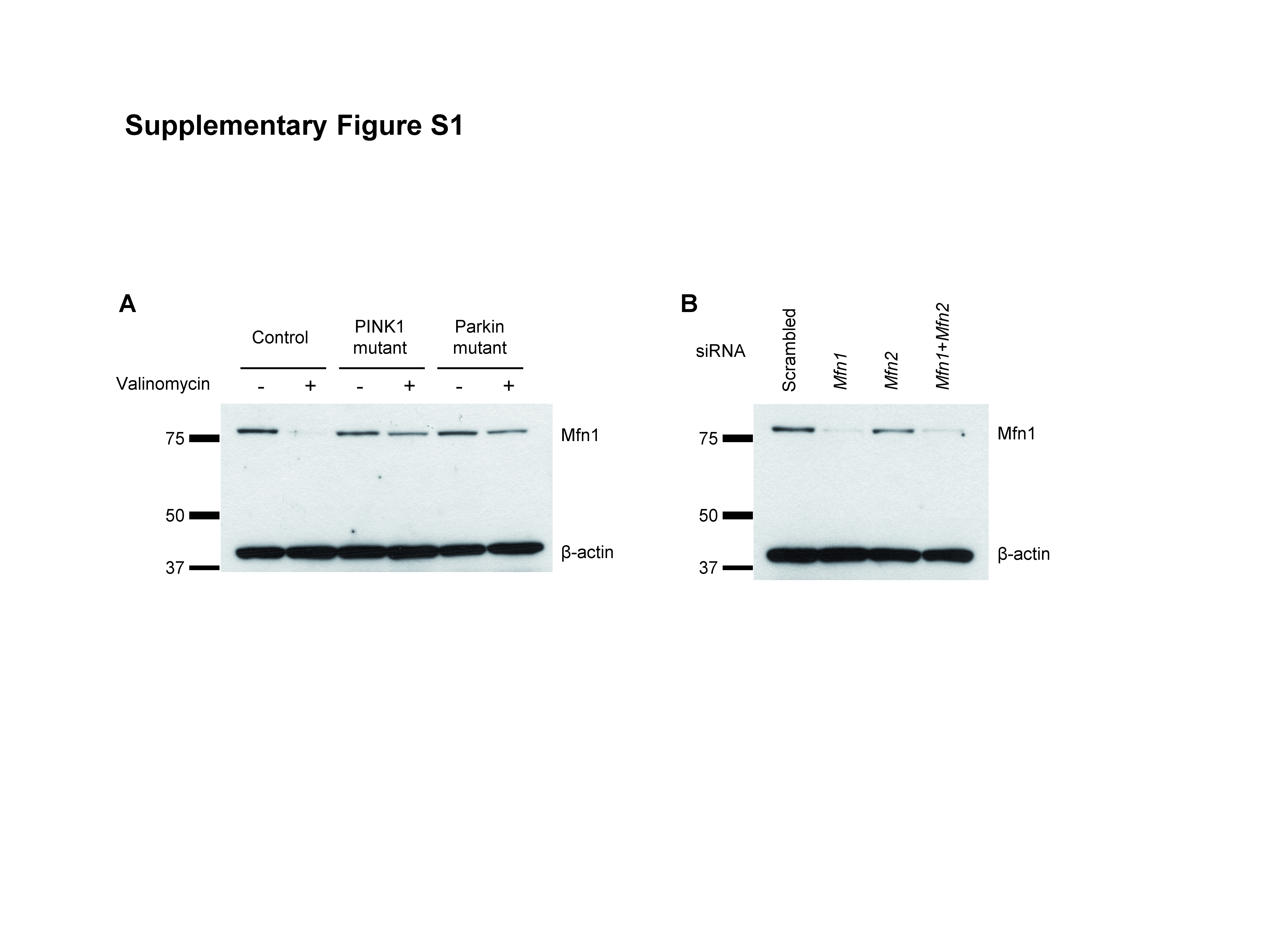

Supplement: Figure S1 — Protein levels of Mfn1 after valinomycin treatment. (A) Fibroblasts from a healthy control, a homozygous PINK1 mutant and a homozygous Parkin mutant were cultured under basal conditions or treated with 1 µM valinomycin for 12 h. The protein levels of Mfn1 were investigated by means of Western blotting. Valinomycin exposure caused a drop in Mfn1 levels in controls, but not in PINK1- or Parkin-mutant cells. β-actin served as a loading control. (B) Mutant cells were transfected with scrambled siRNA, Mfn1 siRNA, Mfn2 siRNA or a combination of Mfn1 and Mfn2 siRNA for 40 h. Western blot analysis was performed with an antibody against Mfn1. The Mfn1 levels decreased only when Mfn1 siRNA was employed, confirming the specificity of the anti-Mfn1 antibody used in our study. Mfn1 – mitofusin 1; Mfn2 – mitofusin 2. (TIFF) [file pone.0016746.s001.tiff]

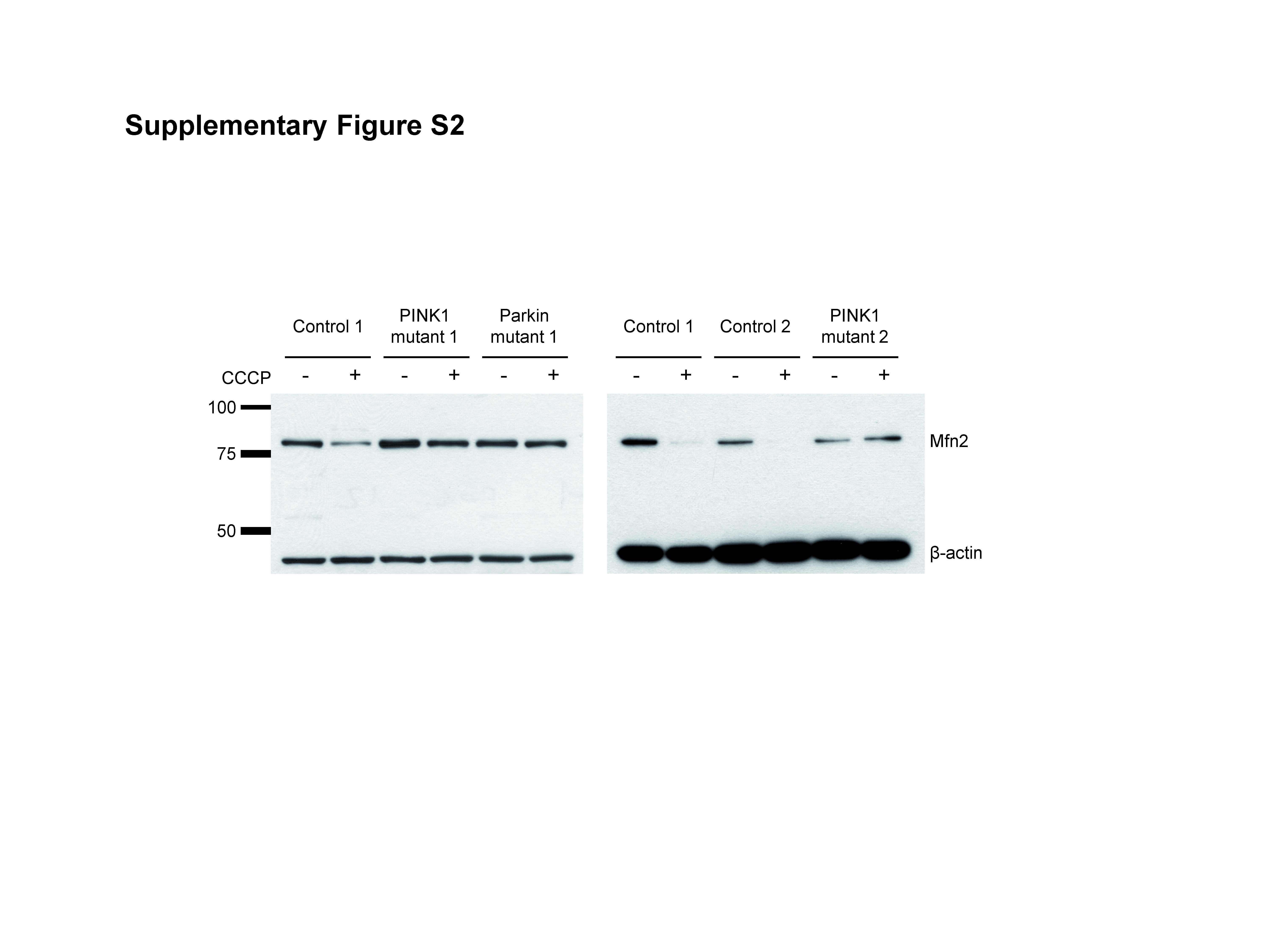

Supplement: Figure S2 — Protein levels of Mfn2 after CCCP treatment. Fibroblasts from two healthy controls, two homozygous PINK1 mutants and one homozygous Parkin mutant were cultured under basal conditions or treated with 10 µM CCCP for 12 h. The protein levels of Mfn2 were investigated by means of Western blotting. CCCP exposure caused a decrease in Mfn2 levels in controls, but not in PINK1- or Parkin-mutant cells. β-actin served as a loading control. CCCP – cyanide m-chlorophenylhydrazone; Mfn2 – mitofusin 2. (TIFF) [file pone.0016746.s002.tiff]

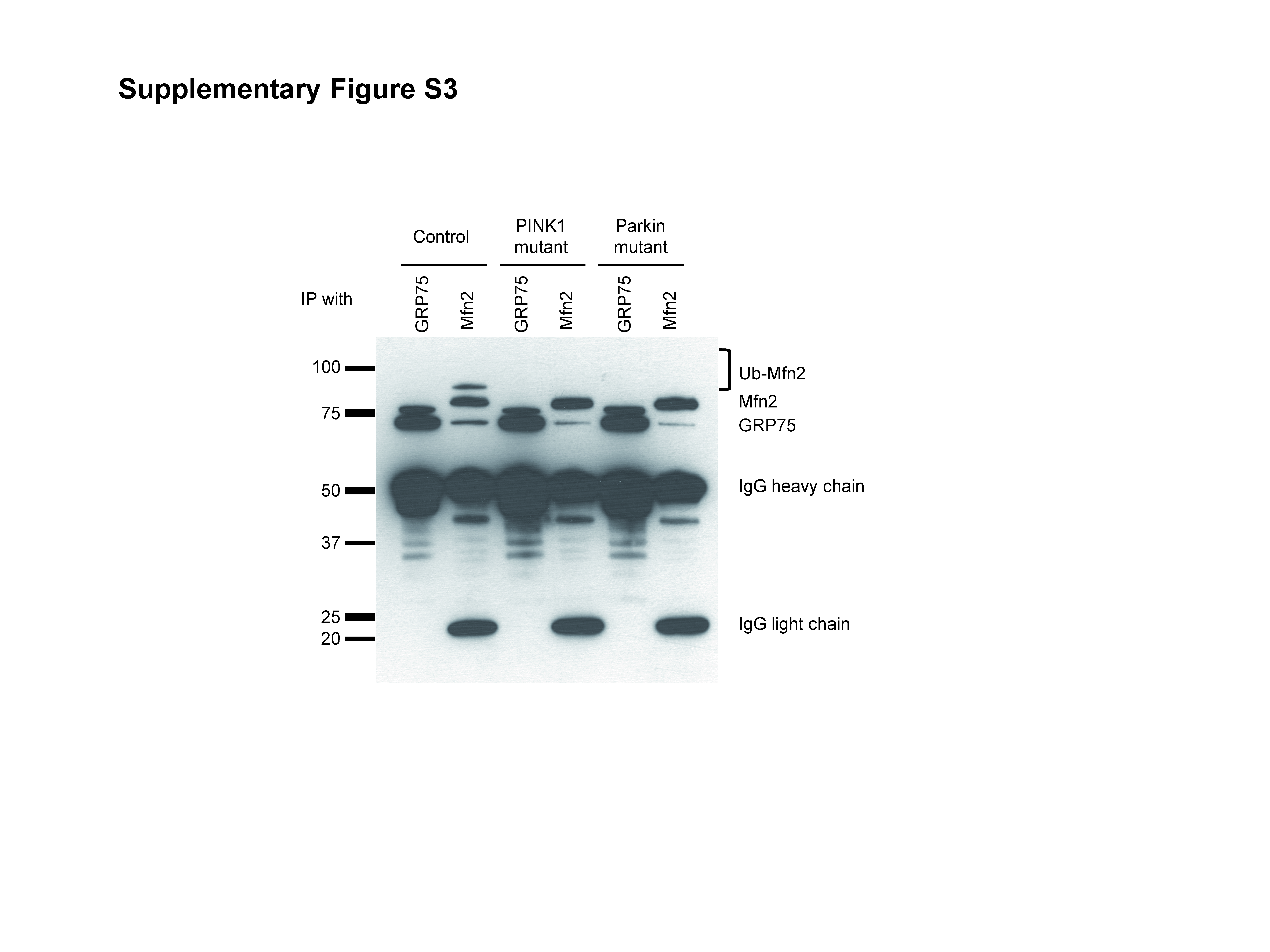

Supplement: Figure S3 — Immunoprecipitation with antibodies against Mfn2. Control, PINK1- and Parkin-mutant cells were treated with 1 µM valinomycin for 12 h. Cells were harvested and whole cell lysates were used for immunoprecipitation with antibodies against GRP75 or Mfn2. The resulting precipitates were analyzed by Western blotting using antibodies against Mfn2 and GRP75. An Mfn2 immunoreactive band of higher molecular weight was detected only in controls but not in either of the mutants. Immunoprecipitation with an antibody against the mitochondrial marker GRP75 served as a negative control. GRP75 – glucose-regulated protein 75; IgG – immunoglobulin G; Mfn2 – mitofusin 2; Ub-Mfn2 – ubiquitylated mitofusin 2. (TIFF) [file pone.0016746.s003.tiff]

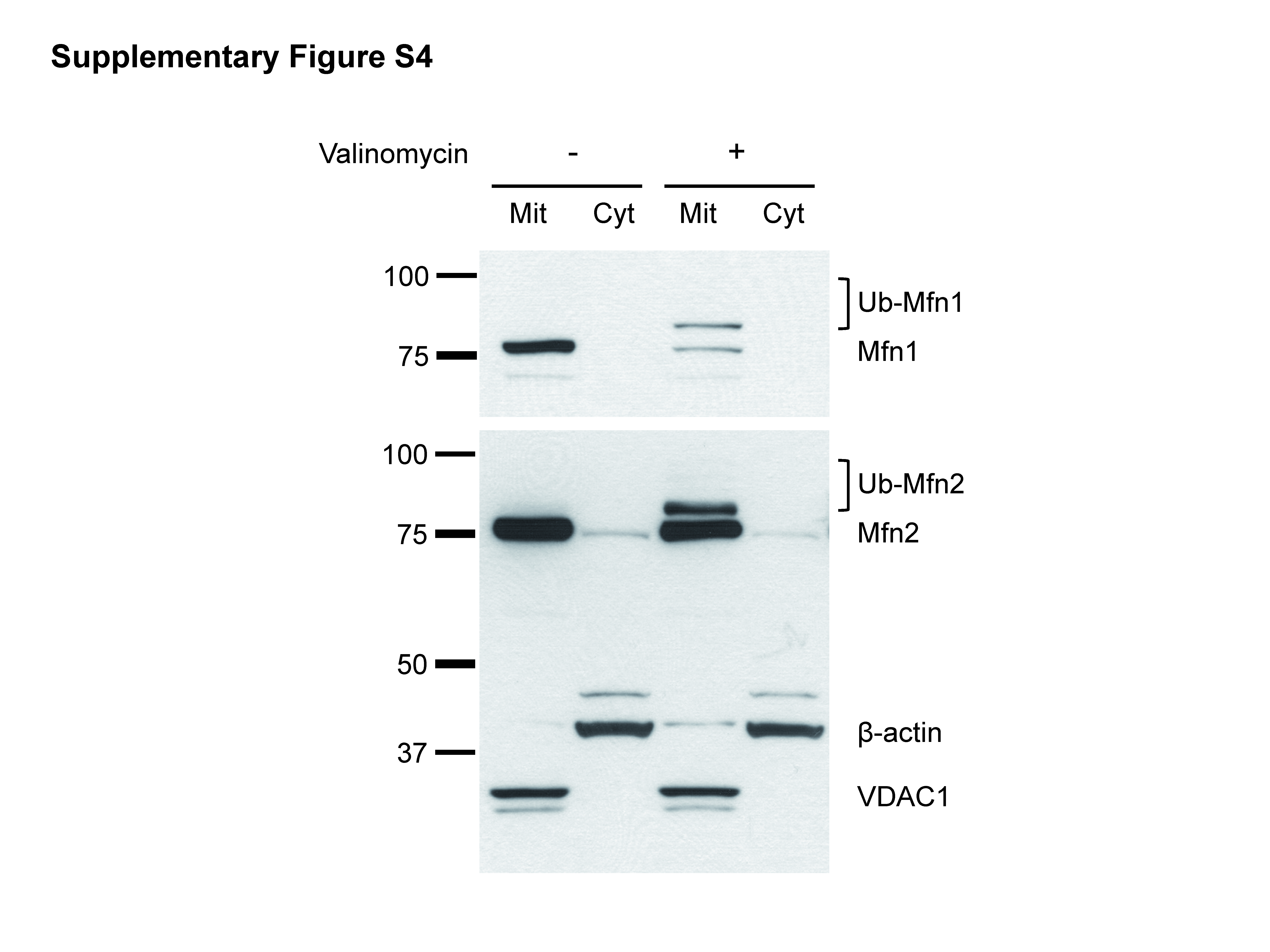

Supplement: Figure S4 — Mitochondrial localization of ubiquitylated Mfn1 and Mfn2 after valinomycin treatment. Control fibroblasts were cultured under basal conditions or treated with 1 µM valinomycin for 6 h. Cells were harvested, proteins of mitochondrial and cytosolic fractions were loaded on two SDS-PAGE gels and analyzed by Western blotting. The subcellular localizations of Mfn1 and Mfn2 were determined. Quality of cellular fractionation was confirmed using antibodies against VDAC1 and β-actin. The ubiquitylated forms of Mfn1 and Mfn2, which were observed only after valinomycin stress, are exclusively found in the mitochondrial fraction. Cyt – cytosolic fraction; Mit – mitochondrial fraction; Mfn1 – mitofusin 1; Mfn2 – mitofusin 2; VDAC1 – voltage-dependent anion channel 1. (TIFF) [file pone.0016746.s004.tiff]

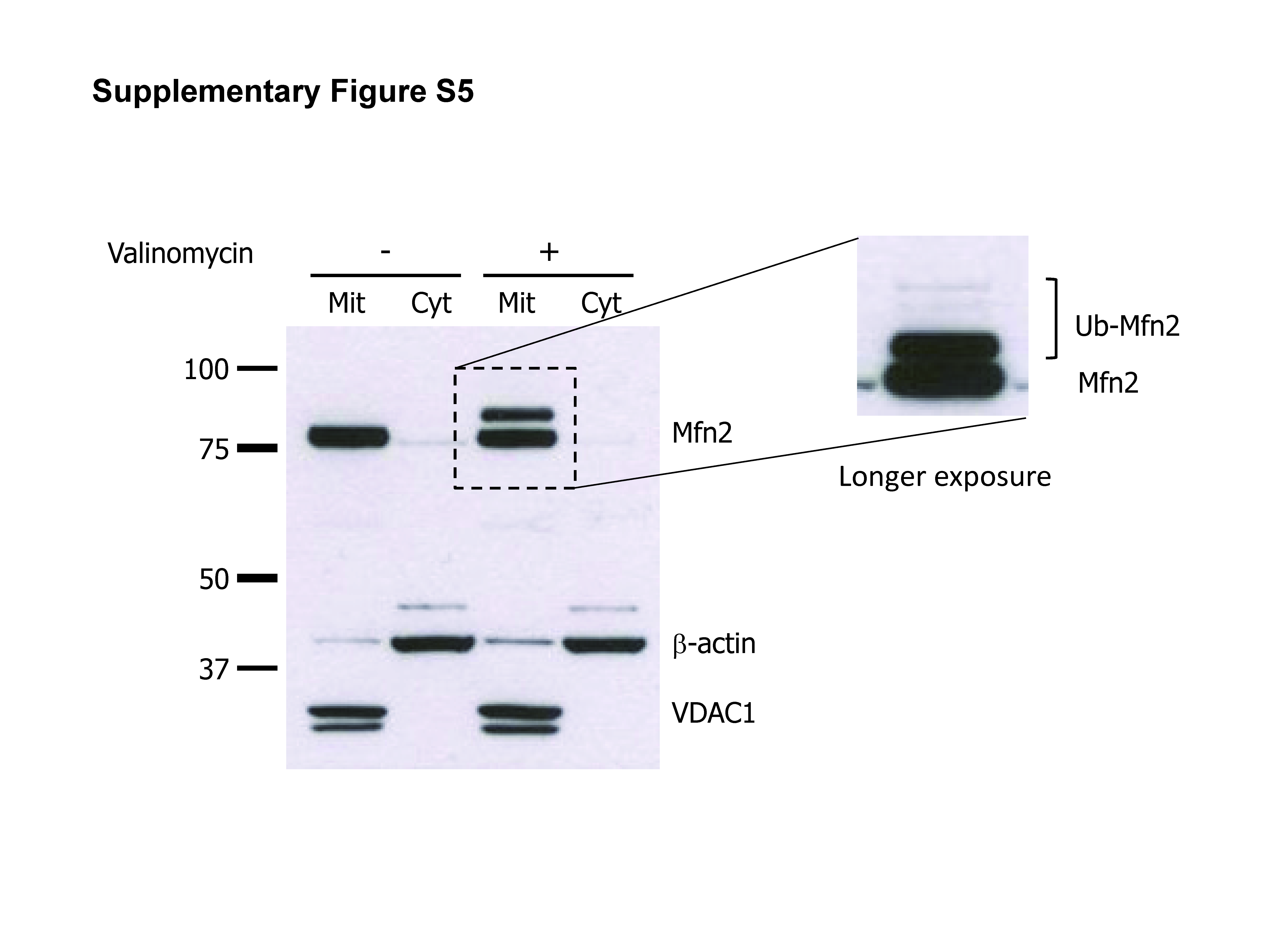

Supplement: Figure S5 — Mitochondrial localization of ubiquitylated Mfn2 after valinomycin treatment in SH-SY5Y cells. SH-SY5Y cells were cultured under basal conditions or treated with valinomycin for 6 h. Cells were harvested and mitochondrial and cytosolic fractions were analyzed by Western blotting. The subcellular localization of Mfn2 was determined. Quality of cellular fractionation was confirmed using antibodies against VDAC and β-actin. The ubiquitylated forms of Mfn2, which were observed only after valinomycin stress, are exclusively found in the mitochondrial fraction. Mfn2 – mitofusin 2; Ub-Mfn2 – ubiquitylated mitofusin 2; VDAC1 – voltage-dependent anion channel 1. (TIFF) [file pone.0016746.s005.tiff]

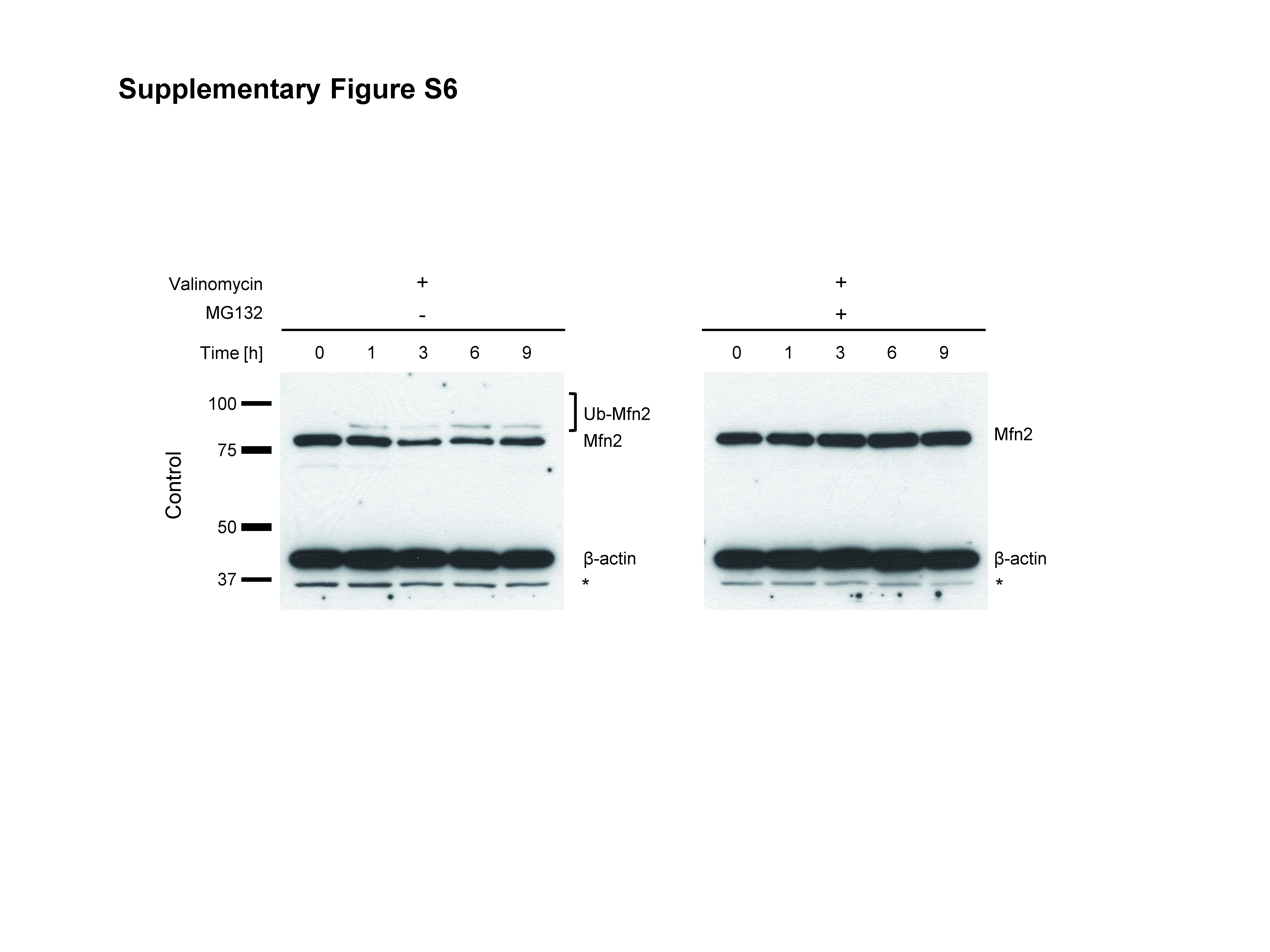

Supplement: Figure S6 — Ubiquitylation of Mfn2 occurs within 1 h of valinomycin treatment. Fibroblasts from a healthy control were treated with 1 µM valinomycin alone (left panel) or with 1 µM valinomycin plus 10 µM MG132 (right panel). Proteins were extracted at different time points and analyzed by Western blotting. Valinomycin treatment initiated the ubiquitylation of Mfn2 after 1 h of incubation. This effect was prevented by simultaneous exposure to MG132. β-actin served as a loading control. An unspecific band is marked by an asterisk. Mfn2 – mitofusin 2; Ub-Mfn2 – ubiquitylated mitofusin 2. (TIFF) [file pone.0016746.s006.tiff]

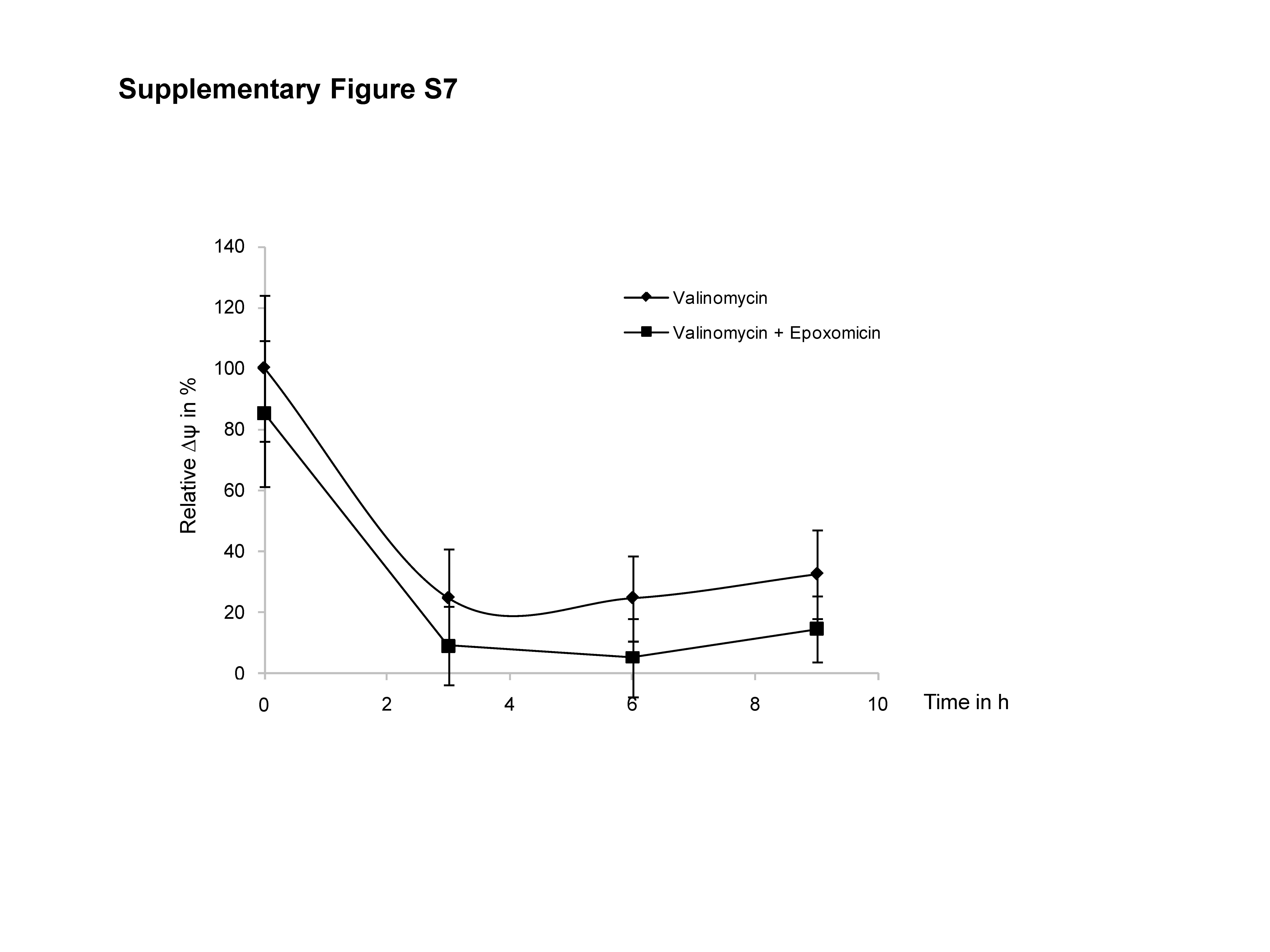

Supplement: Figure S7 — Treatment with valinomycin alone or in combination with epoxomicin causes a drop in mitochondrial membrane potential. Control fibroblasts were incubated with either 1 µM valinomycin alone or with 1 µM valinomycin plus 10 µM epoxomicin. The membrane potential was measured at different time points and corrected for protein concentration. Exposure to the proteasome inhibitor epoxomicin did not influence the membrane potential over time. In the graph mean values +/− standard deviation of three independent experiments are given. Δψ – mitochondrial membrane potential. (TIFF) [file pone.0016746.s007.tiff]

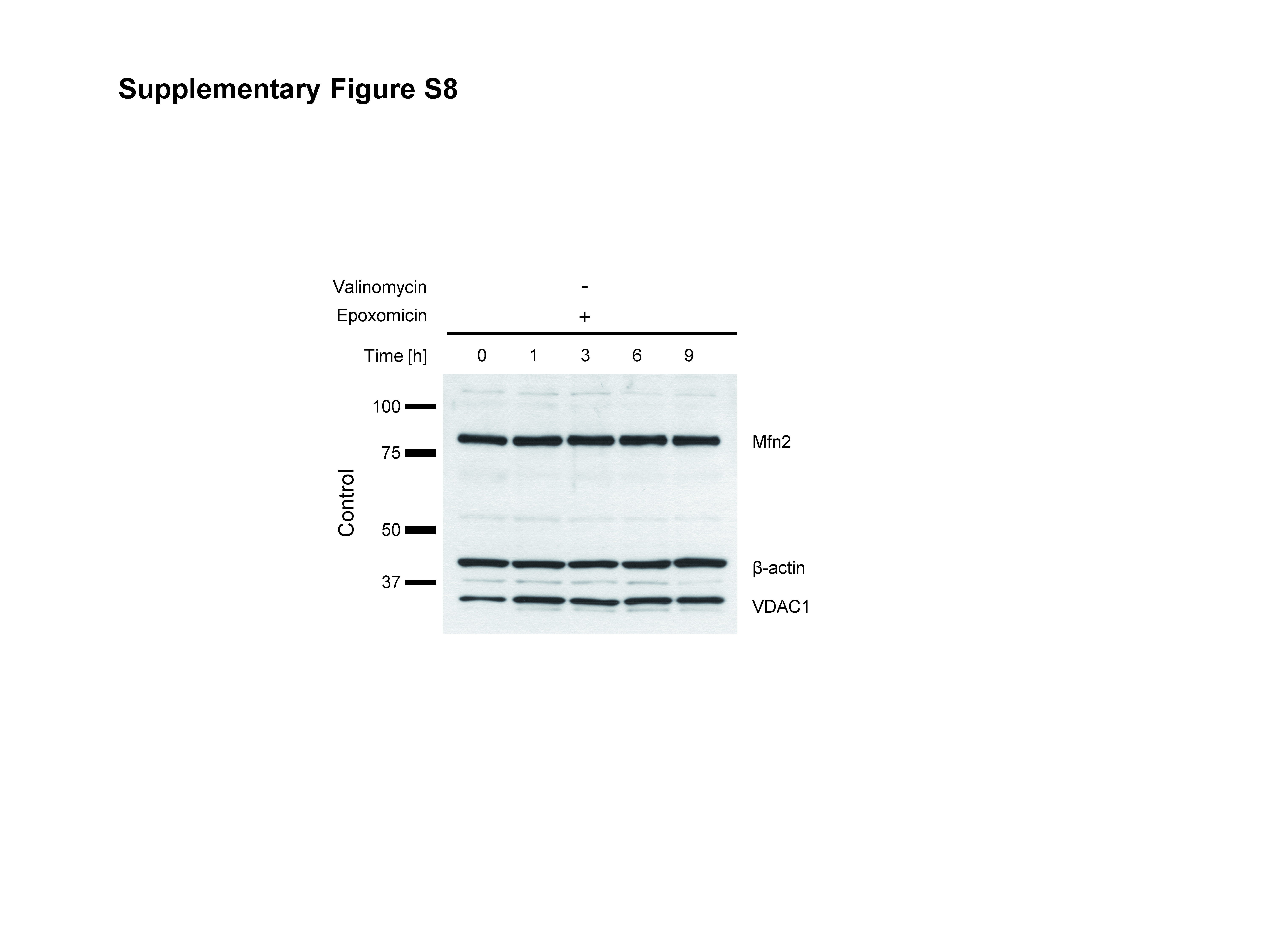

Supplement: Figure S8 — No accumulation of Mfn2 after exposure to epoxomicin. Control fibroblasts were treated with 10 µM epoxomicin. Proteins were extracted at different time points and analyzed by Western blotting. Exposure to the proteasome inhibitor epoxomicin did not affect the expression of Mfn2 over time. The mitochondrial marker VDAC1 and the cytosolic marker β-actin served as loading controls. Mfn2 – mitofusin 2; VDAC1 – voltage-dependent anion channel 1. (TIFF) [file pone.0016746.s008.tiff]
